# Clinical evaluation of giomer- and resin-based fissure sealants on permanent molars affected by molar-incisor hypomineralization: a randomized clinical trial

**DOI:** 10.1186/s12903-022-02298-9

**Published:** 2022-07-05

**Authors:** Beste Özgür, Seren Tuğçe Kargın, Merih Seval Ölmez

**Affiliations:** Department of Pediatric Dentistry, Hacettepe University Faculty of Dentistry, 06100 Altındag, Ankara, Turkey

**Keywords:** Fissure sealant, Giomer, Retention, Molar incisor hypomineralization

## Abstract

**Background:**

Molar-incisor hypomineralization (MIH) is a common condition among children that significantly increases the risk of caries. The objective of this research was to evaluate the clinical success of giomer- and conventional resin-based sealants applied on first permanent molars (FPMs) affected by MIH.

**Methods:**

One-hundred FPMs with MIH which were indicated for non-invasive fissure sealant were selected in 39 children, aged 6–12 years. Using a split mouth design, the FPMs were randomized into two groups; Group 1. Resin sealant (etch-and-rinse + Conceal F) and Group 2. Giomer sealant (self-etch primer + BeautiSealant). Clinical evaluation was performed using the modified United States Public Health Service (USPHS) criteria at 1, 3, 6 and 12 months. The Log-rank, Fisher’s exact test and Kaplan–Meier analysis were used for statistical analysis.

**Results:**

At 12 months, the retention rates in Group 1 and Group 2 were 68% and %8, respectively (*p* = 0.000). The cumulative survival rates of conventional resin sealants were significantly higher than giomer sealants for all follow-up visits (*p* < 0.05). In Groups 1 and 2, the distribution of unsuccessful sealants on mandibular vs maxillary FPMs were 32.1% vs 31.8% (*p* = 0.612) and 91.7% vs 92.3% (*p* = 0.664), respectively. Although the success rate was higher for teeth with white opacities or lesions with less extension in Group 1, no significant difference was found. The average survival time was found as 10.46 ± 3.21 months in Group 1 and 4.02 ± 4.43 months in Group 2.

**Conclusions:**

The conventional resin-based sealants yielded a better clinical performance over the 12-month evaluation period than the giomer sealants which were applied with self-etch primer. The high failure rate observed in giomer sealants could be explained by the possible deficiency in the etching capacity of self-etch primer on MIH-affected teeth.

*Trial registration* ClinicalTrials.gov, NCT04929782. Registered 10 June 2021—Retrospectively registered, https://clinicaltrials.gov/ct2/show/NCT04929782.

**Supplementary Information:**

The online version contains supplementary material available at 10.1186/s12903-022-02298-9.

## Background

Molar incisor hypomineralization (MIH) is a congenital and qualitative enamel defect that primarily involves first permanent molars (FPMs) and frequently affects permanent incisors [1]. The exact prevalence of MIH is not known due to the use of different indexes and diagnostic criteria. Nevertheless a wide prevalence ranging from 2.8% to 44% have been reported in different studies [2].

The disorder is clinically characterized by well-demarcated opacities of varying severity, including the color and extension of the lesion. The defected areas demonstrate lower mineral density and elevated protein/water content, increased porosity, disorganized enamel crystals and enlarged inter-prismatic spaces [3, 4]. This hypomineralized enamel has normal thickness. However, it could be rapidly broken-down following tooth eruption that was defined as post-eruptive breakdown (PEB) [5]. The lesions are mostly observed on the occlusal surface which extend cervically, involving about half of the buccal/lingual surfaces [6].

MIH-affected teeth are more prone to dental caries due the lower mechanical properties (e.g., hardness and modulus of elasticity) of the enamel, PEB and, increased sensitivity during brushing [7–9]. It has been reported that MIH patients had higher caries experience with increased need and frequency of dental treatment [9, 10]. Since MIH poses a high caries risk, an intensive preventive approach should be planned as soon as the lesions are diagnosed on the erupting FPMs [11–13].

Fissure sealants have been suggested for mild cases of MIH where FPMs that do not present PEB but experience increased sensitivity to external stimuli such as air/water [13, 14]. Resin-based sealants are the most commonly used material [15, 16]. Fagrell et al. [17] reported that acid etching of the hypomineralized enamel exposes the organic part of the prism sheaths, which may affect the properties of the restorative material. In 1999, Roberts et al. [18] developed pre-reacted glass ionomer (PRG) filler technology in which fluoroaluminosilicate glass particles that have previously reacted with the polyacrylic acid were dispersed in resin. Based on this and using a bioactive surface pre-reacted glass (S-PRG) filler, a new hybrid material (giomer) that combined the advantages of resin composites with glass ionomer cements has been introduced [19]. The fissure sealants, containing S-PRG filler and bonded by self-etch primer, can maintain the enamel surface integrity without the tags created by acid etching [20]. Although in vitro studies have reported the altered structure of hypomineralized enamel and consequently weaker adhesion with resin restorations [21–23], there has not been enough evidence regarding the survival of different types of sealants on MIH-affected teeth.

The present study aimed to evaluate the clinical success of giomer- and conventional resin-based sealants applied on MIH-affected first permanent molars. The tested null hypotheses were: (1) the clinical success rates of conventional resin-based and giomer-based sealants do not differ, and (2) there is no significant relationship between the clinical features of the MIH lesions (color and extension) and clinical success rates of the sealants used.

## Methods

### Study design

This prospective, non-inferiority, randomized clinical study had a split-mouth design. Its protocol was approved by the Institutional Human Subject Review Committee of Hacettepe University and Ministry of Health Ethics Committee (Protocol No: KA-17130). The localization (buccal, lingual/palatal, occlusal) and the color (white, yellow, brown) of the opacities were recorded by two calibrated clinicians (BÖ, MSÖ) (Additional file 1: Data collection form for lesion characteristics). In case of disagreement, the decision was reached with consensus. The modified United States Public Health Service (USPHS) criteria were used for follow-up evaluations (Table 1) [24]. Two investigators (BÖ, MSÖ), who were blinded to the study groups independently evaluated the sealants and a forced consensus was sought in case of any disagreement. The examiners were calibrated by re-evaluating 10 patients after 1 week. The intra-examiner kappa coefficients for MIH lesions and modified-USPHS Criteria were 0.96 (BÖ)–0.93 (MSÖ) and 0.85 (BÖ)–0.87 (MSÖ), respectively (Cohen’s kappa test).

**Table 1:** The modified United States of Public Health Service Criteria for clinical evaluation of sealants

Category	Score	Characteristic
Anatomic Form	Alpha	Continuous
	Bravo	Slight discontinuity, clinically acceptable
	Charlie	Discontinuous
Marginal Adaptation	Alpha	Closely adapted, no crevice is detected with explorer
	Bravo	Explorer penetrates in no more than 1/3 of the margin
	Charlie	Explorer penetrates more than 1/3 of the margin
Surface Texture	Alpha	Enamel-like surface
	Bravo	Surface rougher than enamel
	Charlie	Surface which unacceptably rough
Marginal Discoloration	Alpha	No visual evidence of discoloration
	Bravo	Discoloration without penetration in pulpal direction
	Charlie	Discoloration with penetration in pulpal direction
Retention	Alpha	No loss of the sealant
	Charlie	Loss of the sealant (partial or total loss)
Secondary Caries	Alpha	No caries present
	Charlie	Caries present

### Selection of participants

The study population comprised healthy children, aged 6–12 years, who presented to the pediatric dentistry clinic at Hacettepe University Faculty of Dentistry for routine dental examination. The diagnosis of MIH was made according to the European Academy of Paediatric Dentistry (EAPD) criteria [25]. The cooperative children who had at least two fully erupted FPMs with occlusal mild MIH defects (demarcated white, yellow or brown lesions), sound surfaces and no incipient enamel caries, where moist control be achieved were invited to the study. The extension of the color change on the occlusal surface (divided into four regions) and the color of the lesion (white, yellow, brown) were recorded indicators of clinical features of the MIH lesions in order to evaluate their effects on fissure sealant success. Children having hypomineralized FPMs with PEB, cavitated carious lesions, restorations, fixed orthodontic appliances or enamel defect(s) due to a condition other than MIH were excluded. The informed and also written consents were obtained from the parent of each participating child.

### Treatment

All fissure sealants were performed by one clinician (STK). The allocation concealment (the material used for the treatment of each tooth) was obtained by using sequentially numbered opaque-sealed envelopes (SNOSEs) that was prepared by an investigator with no clinical involvement in the trial [26]. After cleaning with a bristle brush and a non-fluoridated paste operated by a slow speed hand-piece, the teeth were randomly assigned into two groups by choosing an envelope for each tooth: Group 1. Resin sealant (Conceal F) and Group 2. Giomer sealant (BeautiSealant). A dental isolation device (Mr. Thisty One Step, Zirc Dental, Buffalo, MN, USA) was used throughout the procedures. In Group 1 the occlusal surfaces were etched with 37% phosphoric acid for 30 s (i-GEL N, i-dental, Lithuania), rinsed with air–water spray for 30 s and dried with oil-free air for 15 s. Proper etching was confirmed by a dull frosty‐white appearance of the enamel. The resin sealant (Conceal F, SDI, Australia) applied into the occlusal fissures was light-cured with 460–500 nm wavelength halogen light unit (Hilux Dental Curing Light Unit 250, Benlioğlu Dental Inc, Turkey) for 20 s. In Group 2 the self-etch primer (BeautiSealant Primer, Shofu, Japan) was applied to the occlusal fissures with a fine microbrush. After waiting for 5 s, and homogenizing the bond layer with gentle air stream for 5 s, the giomer sealant (BeautiSealant Paste, Shofu, Japan) was placed and light-cured for 20 s. In both groups, the surfaces were checked with an explorer to ensure that no voids were present. The occlusion was checked and, if necessary, adjusted. The manufacturers’ instructions were followed throughout the application of all materials. Regular oral hygiene instructions were given for all patients and no other caries preventive program was applied.

### Follow-up

Two blinded examiners assessed anatomic form, marginal adaptation, surface texture, marginal discoloration, retention and secondary caries at 1, 3, 6 and 12 months using the modified-USPHS criteria (Table 1) [24]. At each recall, after prophylaxis, teeth were isolated with cotton rolls and air-dried. Fissure sealants were checked under artificial light with a dental mirror and straight explorer. The sealants received “Alpha” or “Bravo” scores from all of the USPHS Criteria (anatomic form, marginal adaptation, surface texture, marginal discoloration, retention and secondary caries) were classified as successful. On the other hand, “Charlie” score from one or more of the USPHS criteria was considered as a failure and these sealants were excluded from further assessment after replacement of the sealant with the conventional technique (etch-and-rinse + resin sealant application). The regular clinical follow-ups of all patients were continued and oral hygiene instructions were given at every recall.

### Statistical analysis

A priori power analysis was performed using G*Power (two-sided z-test, difference between two independent proportions) with 0.05 α error and 0.80 power (1 − β). The success rates of the groups were estimated as 10% and %70 in the light of a similar study [27]. The minimum sample size was reached 13 teeth per group by considering %20 patient attrition rate (drop-out) during 12 months. Statistical analysis was performed using SPSS 20.0 software (IBM Corp., Chicago, IL, USA) and the level of significance was set at *p* < 0.05. The association between independent variable (groups) and dependent variable (USPHS scores) was evaluated with Fisher’s exact test. The crosstabulation of the categorical data regarding the groups were analyzed with chi-square test or Fisher’s exact test. The Log-rank test and Kaplan–Meier analysis were used to calculate and compare the cumulative survival rates of the sealants.

## Results

**Fig. 1:**
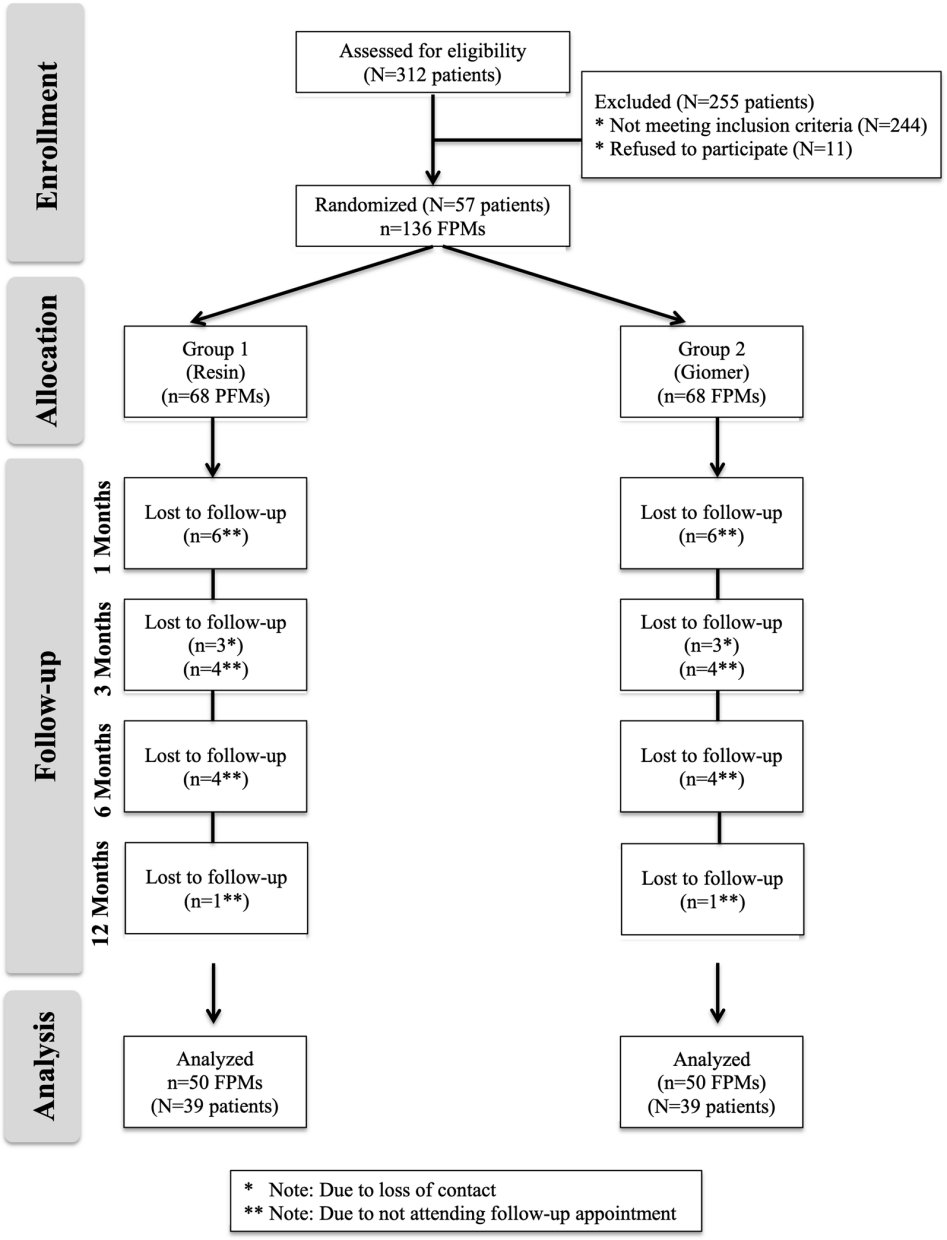
The flow of participants in the study

**Fig. 2:**
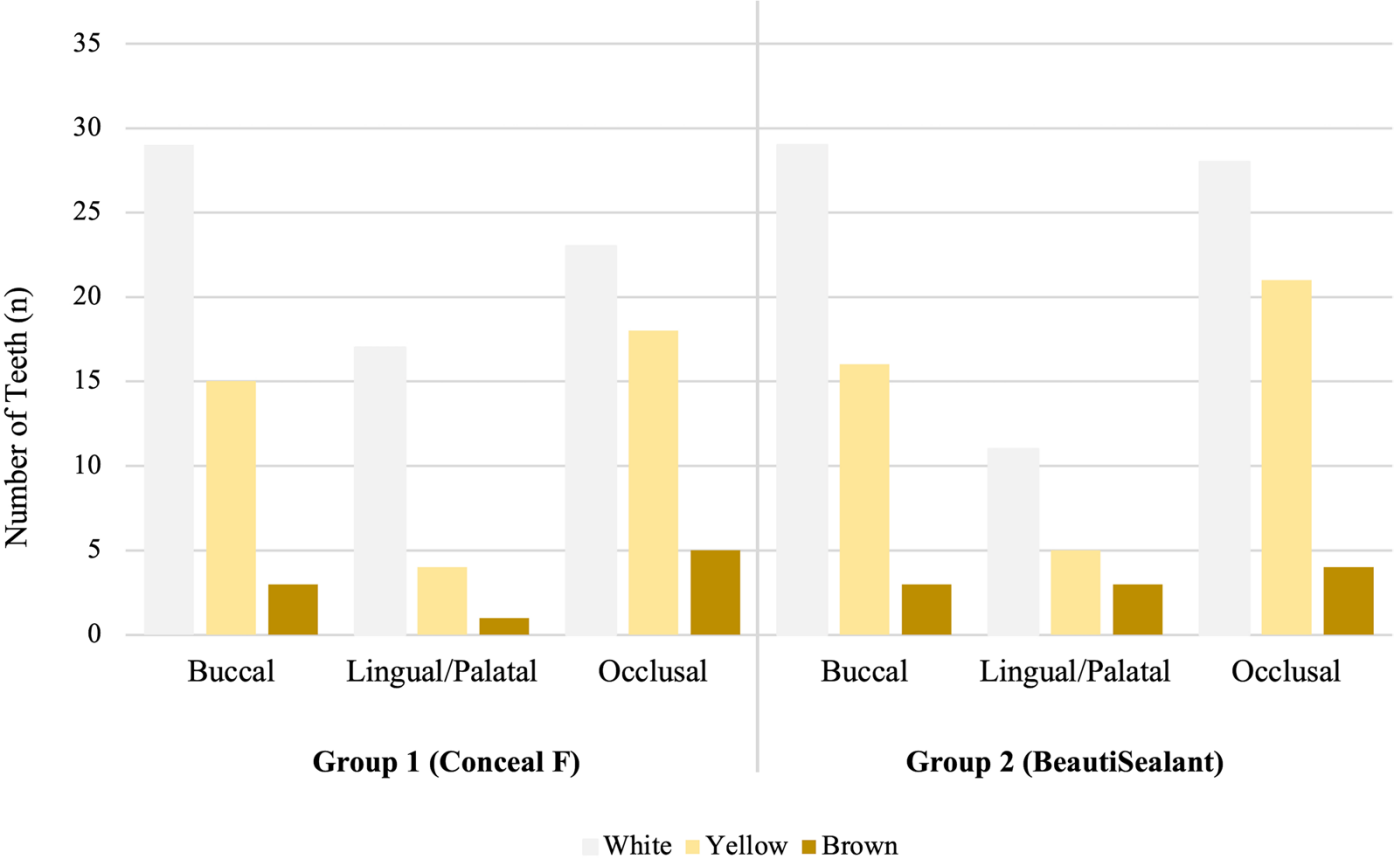
The distribution of MIH lesions (localization and color) regarding the groups

Between January-December 2018, 312 children diagnosed with MIH and 255 did not meet the inclusion criteria (n = 244) or refused to participate (n = 11). Hence, the study was carried out on 57 patients with 136 FPMs. In 11 patients all FPMs were sealed. 18 patients did not return for follow-up, leaving 100 FPMs in 39 patients (13 female, 26 male) for final data analysis (n = 50 teeth per group). The flow diagram of patients is presented in Fig. 1. The mean age of the patients was 8.6 ± 1.4 years. The mean decayed, missing, and filled index for primary teeth (dmft) and permanent teeth (DMFT) were 3.0 ± 2.6 and 0.7 ± 0.8, respectively. At baseline, there was no significant difference between the groups with respect to the number of surfaces (buccal, occlusal, lingual/palatal) affected per tooth (*p* = 0.830). White opacities on all surfaces were more common than yellow or brown lesions and the most affected surfaces were occlusal, followed by buccal and lingual/palatal (Fig. 2).

**Fig. 3:**
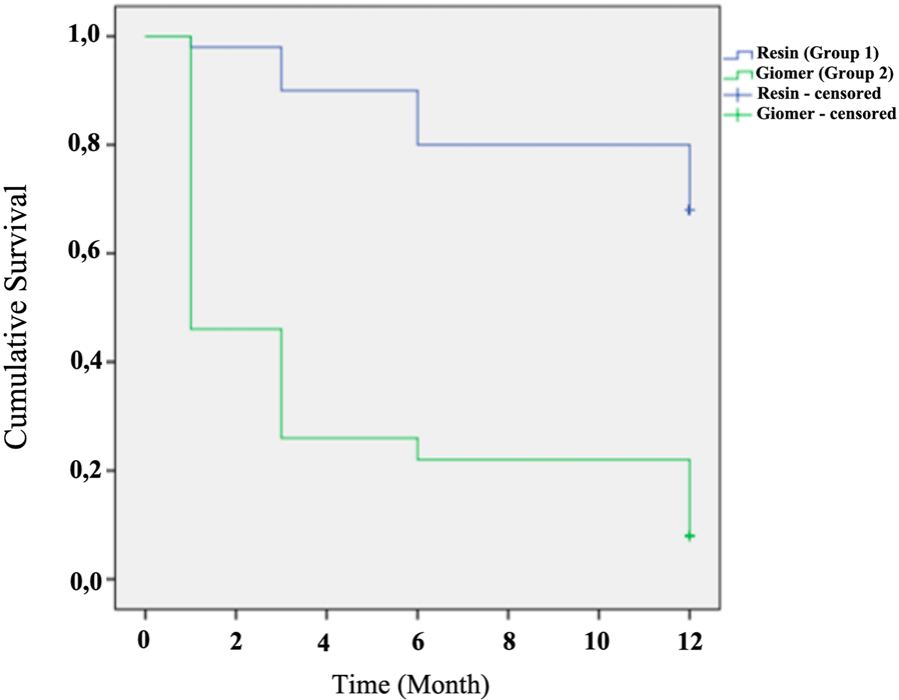
Cumulative survival probabilities of sealants during 12 months (Kaplan–Meier analysis)

After 12 months, the retention rates (score Alpha) in Group 1 (Conceal F) and Group 2 (BeautiSealant) were 68% and %8, respectively (*p* = 0.000). The cumulative survival rates of conventional resin sealants were significantly higher than giomer sealants for all follow-up visits (*p* < 0.05) (Table 2). The Kaplan–Meier survival functions of the groups are presented in Fig. 3. The clinical evaluation of the groups with regard to anatomic form, marginal adaptation, surface texture, marginal discoloration and secondary caries at 1, 3, 6 and 12 months was given in Table 3. None of the sealants received “Charlie” score from these criteria. It was not possible to evaluate the restorations with respect to other USPHS criteria if the sealant was not present (retention score “Charlie”) during follow-ups. Despite the sealant losses, none of the FPMs were observed to develop secondary caries until the last follow-up appointment.

**Table 2:** The survival and failure rates of sealants during the follow-up intervals

	Time (Months)	Number of sealants at the beginning of interval	Retention loss during interval (Charlie)	Failure rate during interval	Survival rate during interval	Cumulative survival rate
Group 1 (Conseal F) N = 50	0–1	50	1	0.020	0.980	0.980*
	1–3	49	4	0.082	0.918	0.900*
	3–6	45	5	0.111	0.889	0.800*
	6–12	40	6	0.150	0.850	0.680*
Group 2 (BS) N = 50	0–1	50	27	0.540	0.460	0.460*
	1–3	23	10	0.435	0.565	0.260*
	3–6	13	2	0.154	0.846	0.220*
	6–12	11	7	0.636	0.364	0.080*

*Log-rank test, *p* < 0.05, BS: BeautiSealant

**Table 3:** Clinical evaluation of the sealants according to anatomic form, marginal adaptation, surface texture, marginal discoloration and secondary caries

Category	Score	Follow-up							
		1-Month		3-Month		6-Month		12-Month	
		Group 1 (Conceal F) No. of teeth	Group 2 (BS) No. of teeth	Group 1 (Conceal F) No. of teeth	Group 2 (BS) No. of teeth	Group 1 (Conceal F) No. of teeth	Group 2 (BS) No. of teeth	Group 1 (Conseal F) No. of teeth	Group 2 (BS) No. of teeth
Anatomic Form	Alpha	42	12	31	6	27	3	23	1
	Bravo	7	11	14	7	13	8	11	3
	Charlie	0	0	0	0	0	0	0	0
Marginal Adaptation	Alpha	41	12	30	5	26	2	22	0
	Bravo	8	11	15	8	14	9	12	4
	Charlie	0	0	0	0	0	0	0	0
Surface Texture	Alpha	48	19	41	11	35	9	31	1
	Bravo	1	4	4	2	5	2	3	3
	Charlie	0	0	0	0	0	0	0	0
Marginal Discoloration	Alpha	49	23	45	13	39	10	33	3
	Bravo	0	0	0	0	1	1	1	1
	Charlie	0	0	0	0	0	0	0	0
Secondary Caries	Alpha	49	23	45	13	40	11	34	4
	Charlie	0	0	0	0	0	0	0	0
Total number of teeth evaluated*		49	23	45	13	40	11	34	4

*If the loss of sealant was determined at the follow-up (retention score = charlie), the restoration could not be evaluated in terms of the other USPHS criteria

In order to evaluate the relationship between clinical features of the MIH lesions and clinical success of the sealants, the occlusal surface was divided into four regions and the affected areas were recorded with lesion color. In Group 1, despite the observed higher clinical success rates when fewer regions were hypomineralized or the dominant lesion color was white, the relationship was statistically insignificant (*p* > 0.05) (Table 4). On the other hand, the comparison did not yield a significant result due to the loss of a large number of sealants in Group 2 (Table 4).

**Table 4:** The effect of the characteristics of the MIH lesion, arches and tooth type on the clinical success of the fissure sealants

Occlusal surface	Group 1 (Conceal F)						Group 2 (BS)					
	Successful (Alpha + Bravo) 12-Month		Unsuccessful (Charlie) 12-Month		Total (Baseline)		Successful (Alpha + Bravo) 12-Month		Unsuccessful (Charlie) 12-Month		Total (Baseline)	
	n	%	n	%	N	%	n	%	n	%	N	%
*Color of the lesion*												
White	24	75.0	8	25.0	32	64.0	2	6.9	27	93.1	29	58.0
Yellow	8	57.1	6	42.9	14	28.0	2	11.8	15	88.2	17	34.0
Brown	2	50.0	2	50	4	8.0	0	0.0	4	100.0	4	8.0
	*p* = 0.549*						*p* = 0.704*					
*Extension of the lesion*												
1/4 of the occlusal	15	78.9	4	21.1	19	38.0	1	5.0	15	95.0	16	32.0
2/4 of the occlusal	7	70.0	3	30.0	10	20.0	0	0.0	13	100.0	13	26.0
3/4 of the occlusal	0	0.0	1	100.0	1	2.0	1	20.0	4	80.0	5	10.0
4/4 of the occlusal	12	60.0	8	40.0	20	40.0	2	12.5	14	87.5	16	32.0
	*p* = 0.374*						*p* = 0.447*					
*Arch*												
Upper	15	62.8	7	31.8	22	44.0	2	7.7	24	92.3	26	52.0
Lower	19	67.9	9	32.1	28	56.0	2	8.3	22	91.7	24	48.0
	*p* = 0.612*						*p* = 0.664*					
*Tooth type*												
16	8	61.5	5	38.5	13	26.0	0	0.0	12	100.0	12	24.0
26	7	77.8	2	22.2	9	18.0	2	14.3	12	85.7	14	28.0
36	11	84.6	2	15.4	13	26.0	2	16.7	10	83.3	12	24.0
46	8	53.3	7	46.7	15	30.0	0	0.0	12	100.0	12	24.0
	*p* = 0.287*						*p* = 0.255*					
Total	34	68.0	16	32.0	50	100	4	8.0	46	92.0	50	100

*Fisher’s exact test, BS: BeautiSealant

In Groups 1 and 2, the distribution of unsuccessful sealants on mandibular vs maxillary FPMs were 32.1% vs 31.8% (*p* = 0.612) and 91.7% vs 92.3% (*p* = 0.664), respectively (Table 4). No significant relationship was observed in both groups regarding the arches. The clinical success in Group 1 (Conceal F) was significantly higher for both maxillary (*p* = 0.000) and mandibular teeth (*p* = 0.000). For each group, there were no statistically significant differences between the failure rates of sealants according to the tooth type (*p* = 0.287 for Group 1 and *p* = 0.255 for Group 2) (Table 4). The average survival time was found as 10.46 ± 3.21 months in Group 1 and 4.02 ± 4.43 months in Group 2.

## Discussion

Several studies reported that children with MIH presented higher caries experience in permanent dentition and received dental treatment for their FPMs nearly 10 times more often than unaffected children [9, 28, 29]. Without preventative care, hypomineralized teeth are at high risk of PEB or dental caries [13]. Fissure sealants may offer a treatment approach for cases where only opacities are present without enamel loss [13, 30]. The literature is very limited about the use of sealants on MIH-affected teeth and more clinical trials are required to identify appropriate restorative alternatives. The present study compared the clinical survival probabilities of giomer- and conventional resin-based sealants on first permanent molars with MIH.

The adhesion of resin materials to hypomineralized enamel were significantly lower than unaffected teeth [22, 31]. Even in the normal looking enamel of a MIH-affected tooth, less demineralization in the inter-prismatic spaces and exposure of rounded end rods (type III) do not correspond to the regular phosphoric acid etching pattern [32]. Therefore, it is improper for achieving a good adhesion of restorative material. A clinical study by Lygidakis et al. [33] showed that 21% of the resin sealants, which were placed with conventional technique (etch-and-rinse + resin sealant application) with prior mechanical preparation, were lost (partially or total) 12 months after placement. On the other hand, a higher retention rate was achieved after four years by using a 5^th^ generation bonding agent as an intermediate layer [33]. Fragelli et al. [34] compared the survival rates of resin sealants applied to the sound and MIH-affected molars without mechanical preparation or adhesive application. After 1 year, the authors reported 37.4% and 24% sealant loss for sound and hypomineralized teeth, respectively. They related their findings to possible remineralization of teeth which was achieved by four-time weekly application of fluoride varnish prior to the treatment [34]. In the present study no mechanical preparation of the fissures or pretreatment with fluoride varnish was performed. These might have contributed to the relatively higher annual failure rate observed for the conventional resin sealants (32%).

Giomer sealants have been shown to prevent demineralization, microleakage and gap formation without microretention in tags obtained by acid etching [20]. An in-vitro study by Shimazu et al. [20] reported that giomer and resin sealants have similar enamel shear bond strength values on different enamel conditions (sound or subsurface lesion). On the other hand, Özer et al. [35] presented that the shear bond strengths of giomer sealants (BeautiSealant), placed on sound enamel with acid etching and primer application, were lower than those applied without etching and also lower than conventional resin sealants. Although the self-etching primer has been shown to etch hypomineralized surface deeper than sound enamel, the significantly higher bond strengths achieved for sound enamel suggested other factors rather than micromechanical were also involved in adhesion [22].

The present study was the first to evaluate the clinical performance of giomer and conventional resin sealants on MIH-affected molars. Nearly all giomer sealants (46 out of 50) were lost at the end of 12 months. In a clinical study performed on sound molars, using the same clinical steps of the present study, partial and total retention loss of the giomer (BeautiSealant) and resin sealants (Helioseal F) after 12 months was found to be 52.2% and 39.1%, respectively (*p* = 0.006) [36]. Recent clinical trials also reported poor retention rates (82.9% and 93.1% for partial and total loss) for the giomer sealant (BeautiSealant) on sound molars after 18 months [27, 37]. The self-etch adhesive systems have been recommended in children as they require fewer treatment steps and decrease the patient-related technique sensitivity [38]. However, their bonding efficacy is much controversial even for unground sound enamel, since most of these agents are not as acidic as phosphoric acid [39, 40]. A systematic review and meta-analysis showed that the lower bond strengths due to the lesser ability of self-etch adhesives to penetrate the prisimless outermost layer of enamel may be associated with the poor sealant retention compared with etch-and-rinse systems [41]. Ataol et al. [42] reported that the absence of 10-Methacryloyloxydecyl Dihydrogen Phosphate (MDP) monomer, which improves adhesion by binding to calcium hydroxyapatite and forms 10-MDP-Calcium salts with low solubility, in the one-step self-etch adhesive (BeautiSealant primer) may be related to the lower sealing effectiveness compared to universal adhesives. The high failure rate observed in giomer sealants could be explained by the possible deficiency in the etching capacity of self-etch primer on MIH-affected teeth. Thus, the first null hypothesis was rejected.

Fissure sealants with surface reaction-type PRG fillers (giomer) and bonded by self-etch primer can inhibit caries by improved fluoride-releasing and -recharging properties [43, 44]. During the follow-up period of the present study, no secondary caries was observed in any of groups. This finding was in accordance with the studies which reported no significant difference in caries prevention performance between the conventional resin and the S-PRG filler-containing sealants [27, 36]. Despite the high retention loss of the giomer sealant, it was assumed that the S-PRG particles remained in the fissures could have prevented the development of caries lesions [27].

The color of hypomineralized lesions is a sign for alterations in hardness, porosity and mineral content [6, 30]. Yellow–brown defects which extend through the full thickness of enamel have greater porosity, lower mineral density and lower mechanical strength than white-cream opacities [3, 6]. In a later study, which conventional resin sealants were placed on MIH-affected molars (n = 21), 4 of 7 teeth with unsuccessful sealants had brown and 3 had yellow lesions, while no failure was recorded for white opacities [34]. In the present study, detailed data were recorded in order to evaluate the effect of MIH lesion characteristics on sealant success. Although the success rate was higher for teeth with white opacities or lesions with less extension (1/4 of the occlusal surface) in Group 1, no significant difference was found due to the sample size and nonhomogeneous distribution of the subgroups. It was also not possible to discuss the related finding in Group 2 as almost all sealants were lost. Consequently, the second null hypothesis was accepted.

The relatively small sample size (n = 50 teeth/group) and limited follow-up period have to be stated as the limitations of the present study. According to the World Dental Federation (FDI), dental caries prevalence in 6–19-year-old children in Turkey is over 80 percent [45]. The study was carried out with a split-mouth design which allowed for better control of patient-related factors such as oral hygiene and diet. However, the effort to fulfill the inclusion criteria of having two MIH-affected FPMs free of PEB and indicated for conventional fissure sealant in a high caries risk population resulted in a limited sample size. Furthermore, each of the FPMs affected by MIH may have a different structural, mechanical or chemical presentation despite the split-mouth design and could also be considered as a limitation. In addition, a longer evaluation period was initially planned, but the trial had to be terminated after 12 months since almost all giomer sealants were lost (Group 2). Nevertheless, the present study is the first to assess the clinical success of conventional resin-based and giomer fissure sealants on MIH-affected FPMs.

## Conclusions

The conventional resin-based sealants (Conceal F) yielded a better clinical performance over the 12-month evaluation period than the giomer sealants (BeautiSealant) which were applied with self-etch primer. The color or extension of the MIH lesion had no significant effect on the survival of sealants. However, the retention rate of the conventional resin-based sealant was slightly higher for FMPs with white opacities or less extension (1/4 of the occlusal surface).

## Supplementary Information

**Additional file 1:** Data collection form for lesion characteristics.

## Data Availability

The data that support the findings of this study are available from the corresponding author upon reasonable request.

## Abbreviations

MIH: Molar incisor hypomineralization
FPMs: First permanent molars
USPHS: United States Public Health Service
PEB: Post-eruptive breakdown
PRG: Pre-reacted glass ionomer
S-PRG: Surface pre-reacted glass
EAPD: European Academy of Paediatric Dentistry
SNOSEs: Sequentially numbered opaque-sealed envelopes
BS: BeautiSealant
FDI: World Dental Federation
