# Supplementary figures and images for: Clinical evaluation of giomer- and resin-based fissure sealants on permanent molars affected by molar-incisor hypomineralization: a randomized clinical trial
Source: BMC Oral Health. 2022 Jul 5;22:275. doi: 10.1186/s12903-022-02298-9 (PMC9258125; doi:10.1186/s12903-022-02298-9)

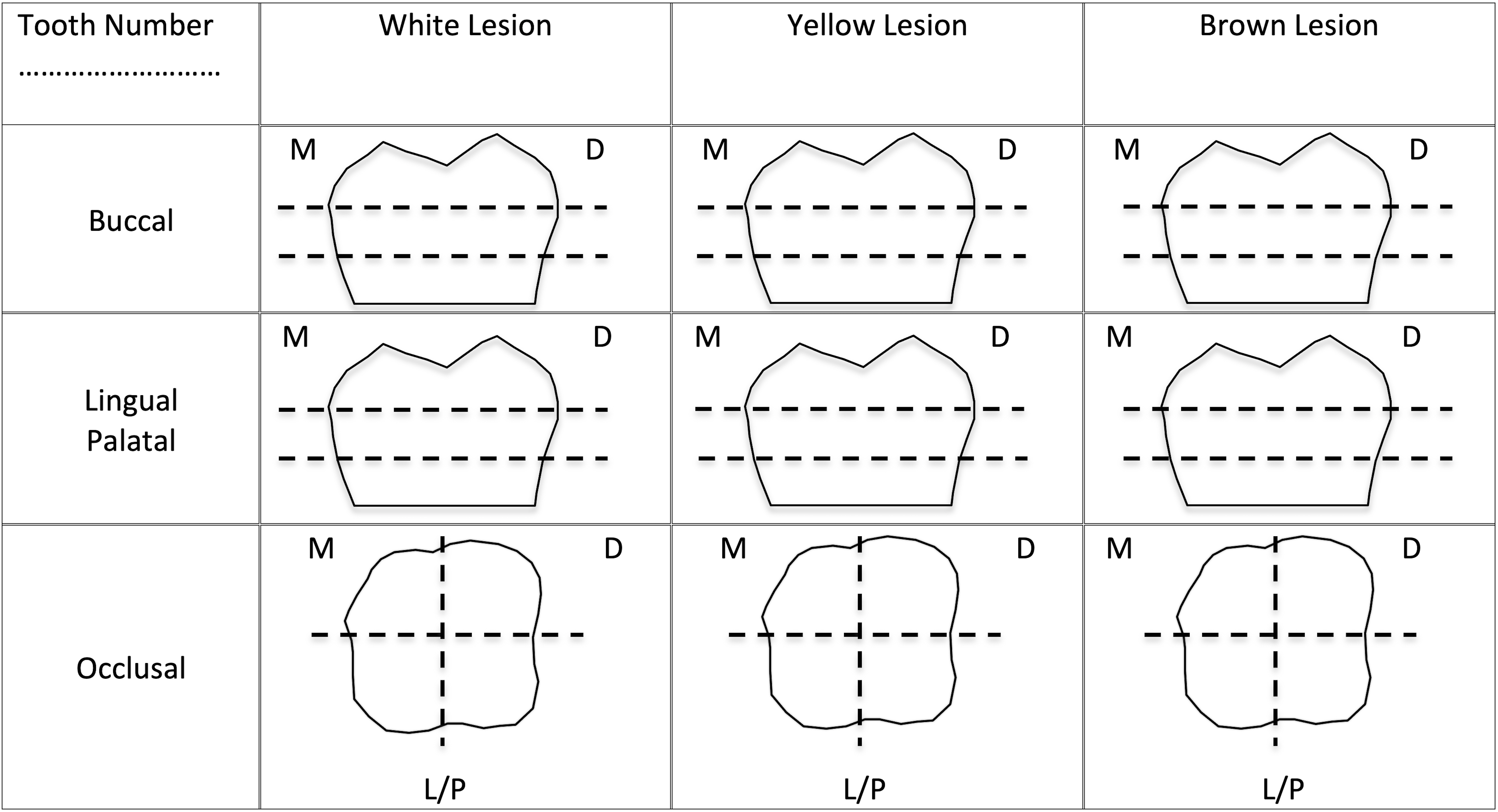

Supplement: Supplementary file 1 — Additional file 1: Data collection form for lesion characteristics. [file 12903_2022_2298_MOESM1_ESM.jpg]
